# ASSOCIATION BETWEEN FRAILTY AND DEVELOPMENT OF COGNITIVE IMPAIRMENT: EVIDENCE FROM THE HEALTH AND RETIREMENT STUDY

**DOI:** 10.1093/geroni/igz038.2521

**Published:** 2019-11-08

**Authors:** Nicholas V Resciniti, Jaleel McNiel, Matthew Lohman

**Affiliations:** University of South Carolina, Columbia, South Carolina, United States

## Abstract

Research has shown there is currently an increasing prevalence of cognitive impairment and dementia in older adults. To date, there remains a paucity of research to explain this increase and research on early markers and risk factors are warranted. This study aims to assess the association of cognitively normal older adults who are frail and the development of cognitive impairment four years later. Data from the Health and Retirement Study – a nationally representative sample of older US adults – was used from 2004-2008 for individuals 65 and older (n=8,377). Frailty was categorized by using Fried’s phenotype model: individuals were grouped into frail, pre-frail, and robust. Cognitive impairment – a composite score that assessed memory recall and global mental status – was classified as scoring eight or less on a 35-point scale. After restricting to cognitively healthy individuals, logistic regression with weights was used to assess the association between frailty status and the development of cognitive impairment four years later. The model was adjusted for baseline age, gender, race, education years, smoking status, and chronic health issues (high blood pressure, diabetes, cancer, lung disease, heart disease, stroke, psychiatric problems, and arthritis). Frail individuals, compared to those who were robust, had increased odds of cognitive impairment (OR=1.74; 95% CI: 1.48-2.16), after fully adjusting. Evidence from this study suggest that frail individuals are more likely to become cognitively impaired over time. This provides a potential pathway of intervention to help delay or prevent the development of cognitive impairment in older US adults.

